# Development of a community health workers perceptual and behavioral competency scale for preventing non-communicable diseases (COCS-N) in Japan

**DOI:** 10.1186/s12889-022-13779-5

**Published:** 2022-07-26

**Authors:** Yuki Imamatsu, Etsuko Tadaka

**Affiliations:** 1 Faculty of Nursing, SOKA University, 1-236 Tangi-machi, Hachioji, 192-8577 Tokyo, Japan; 2 Department of Community and Public Health Nursing, Graduate School of Health Sciences and Faculty of Medicine, Hokkaido University, N12-W5, Kita-ku, Sapporo, 060-0812 Japan

**Keywords:** Community health workers, Competency, Non-communicable diseases, Scale development

## Abstract

**Background:**

Community health workers in Japan are commissioned to work on a voluntary basis on behalf of their communities, to promote healthy behaviors. They are a valuable resource because they can often provide health information and services for local residents with whom professionals find it difficult to engage. However, no instruments exist for evaluating perceptual and behavioral competencies for prevention of non-communicable diseases among voluntary unpaid community health workers in developed countries. This study aimed to develop a community health workers perceptual and behavioral competency scale for preventing non-communicable diseases (COCS-N), and to assess its reliability and validity.

**Methods:**

We conducted a cross-sectional study using a self-reported questionnaire. A total of 6480 community health workers across 94 local governments in Japan were eligible to participate. We evaluated the construct validity of the COCS-N using confirmatory factor analysis, and assessed internal consistency using Cronbach’s alpha. We used the European Health Literacy Survey Questionnaire and the Community Commitment Scale to assess the criterion-related validity of the COCS-N.

**Results:**

In total, we received 3140 valid responses. The confirmatory factor analysis identified eight items from two domains, with perceptions covered by “Sharing the pleasure of living a healthy life” and behavioral aspects by “Creating healthy resources” (goodness-of-fit index = 0.991, adjusted goodness-of-fit index = 0.983, comparative fit index = 0.993, root mean square error of approximation = 0.036). Cronbach’s alpha was 0.88. COCS-N scores were correlated with European Health Literacy Survey Questionnaire scores and Community Commitment Scale scores (*r* = 0.577, *P* < 0.001 and *r* = 0.447, *P* < 0.001).

**Conclusions:**

The COCS-N is a brief, easy-to-administer instrument that is reliable and valid for community health workers. This study will therefore enable the assessment and identification of community health workers whose perceptual and behavioral competency could be improved through training and activities. Longitudinal research is needed to verify the predictive value of the COCS-N, and to apply it to a broader range of participants in a wider range of settings.

**Supplementary Information:**

The online version contains supplementary material available at 10.1186/s12889-022-13779-5.

## Background

Non-communicable diseases (NCDs), also known as chronic diseases, have a long duration and result from a combination of genetic, physiological, environmental and behavioral factors [1, 2]. In 2016, according to a report by an international expert organization, there were 523 million people globally with cardiovascular disease, 463 million with diabetes, and 40 million who had experienced a stroke [3–5]. The incidence of cardiovascular disease has doubled in the past 30 years and that of diabetes has more than tripled in the past 10 years [3, 4]. Globally, approximately 671 million people are presently considered obese (a major primary risk factor for NCDs), which is 2.5 times the number recorded 30 years ago [6]. NCDs often result in sequelae that, on their own, can interfere with daily life and increase the risk of requiring nursing care [7]. However, early primary prevention for NCDs and their major risk factors (tobacco use, physical inactivity, harmful use of alcohol, and unhealthy diets and lifestyle) can lead to longer and healthier lives. Preventing NCDs and extending healthy life expectancy lead to improved quality of life for individuals and better societal outcomes. It is therefore essential to address national NCD prevention and control because of the rising global morbidity and mortality from this group of diseases.

Many national programs for NCD prevention and control have a strong community component. Community health workers (CHWs) are increasingly being recruited to support NCD prevention at a regional level. CHWs are known by many names internationally, including community health agents, community health assistants, and health advisors [8, 9]. CHWs are usually members of the communities in which they work, who are selected by their communities, answerable to those communities for their activities, and supported by the healthcare system [10].

CHWs have four main roles and functions in NCD prevention and control: health education, social support, advocacy, and coordination. Health education is used to increase patients’ and community members’ knowledge and help reduce NCDs and their major risk factors. Social support can be emotional, appraisal (providing information to support self-evaluation), informational, or material. Advocacy and coordination are focused on supporting residents’ access to healthcare institutions and community health professionals, and acting as a bridge for collaboration among CHWs themselves [11, 12]. Previous studies of CHWs have focused mainly on developing countries, and many studies have not examined NCDs. In developing countries, in light of critical shortages in healthcare resources, CHWs often constitute the backbone of primary healthcare services [13]. CHWs are cost-effective in comparison to other parts of the healthcare system [14] and effective for delivering essential maternal and child health, family planning, and nutrition services in developing countries [15, 16]. In developed countries, including Japan, CHWs may also be a useful element of primary healthcare services, particularly in view of recent increases in health inequalities. CHWs may be particularly effective in delivering NCD prevention and control services.

Japan had a human development index score of 0.903 in 2016 (ranked 17th in the world), and has an exemplary record in human development [17]. Every citizen in Japan has had access to universal health insurance since 1960, which means that all citizens have access to medical services without financial barriers. This has contributed to approximately equal access to healthcare and relatively small disparities in health status across regions and socio-economic groups [18]. Despite earlier achievements, however, health inequity is now increasing, and has become a challenge for Japan. The gap between the Japanese prefectures with the lowest and highest life expectancies widened from 2.5 years in 1990 to 3.1 years in 2015, and there is concern that the gap in healthy life expectancy is also widening [19]. Factors determining health inequalities are thought to involve physical and cognitive habits caused by early life experiences, and the surrounding environment (resources for physical and human health) [19]. Many of the prefectures with the highest healthy life expectancy rankings were found to have a high percentage of municipalities with a high level of financial support for healthcare activities, including CHWs. This financial support was not necessarily related to the tax revenue of the prefecture or municipality [20–22]. It is therefore an opportune time to examine approaches to transforming people’s perceptions and identifying human and material resources to reduce health inequalities, to help achieve the Sustainable Development Goal of “leaving no one behind”, so that good health is accessible to all [23]. CHWs can contribute to addressing this issue.

CHWs in Japan are local residents, called community/local health promoters, who are commissioned by national and local public health organizations. There have been CHWs in Japan since the 1950s. CHWs in Japan are typically unpaid volunteers, but the provision of expenses for their activities varies from prefecture to prefecture. CHWs in Japan participate in training programs organized by local governments, either by volunteering or by being recommended by local residents. Many of the applicants are stay-at-home parents whose children have left home, or retirees who join the program to use their leisure time to improve their own and their community’s health. They have a key role in changing perceptual and behavioral competencies to help prevent NCDs. The content of the training program varies slightly between municipalities, but generally lasts between 1 and 6 months, and includes lectures on the pathology of NCDs and related lifestyle habits, using diet to prevent NCDs, easy exercises and local resources that can be used for exercise [24]. CHWs cover the intersection between local residents and local government, and offer activities at the individual, interpersonal, group, and community levels. For example, the role of CHWs can range from introducing recipes for low-salt and low-sugar meals to prevent NCDs, through explaining how to do weight training at home, to introducing health programs at health centers, such as health screening in communities with high blood pressure and diabetes. The most important competence among CHWs is to be able to help community members to review and improve their health Perceptions and Behaviors based on basic knowledge and skills about the prevention of NCDs [25]. However, it is not yet clear which competencies among CHWs contribute to positive health outcomes. Currently, the biggest challenges in community health internationally center around equitable payment for labor, integrating the work of CHWs into health systems without distracting from or disrupting their positions in communities, and combining CHWs’ inputs with other material social support to produce better outcomes with even greater returns on investment. Overcoming these challenges will require a variety of inputs, and each will be different depending on the context. To date, the scales used to measure the competence of CHWs have generally been adapted from scales for staff requiring medical expertise, although a competency scale for community health staff has been developed [26]. Measures for volunteers working in developed countries have been developed to measure motivation [27, 28], but not competencies such as perceptions and behaviors. Japanese CHWs are unique in the sense that they are using their leisure time to prevent NCDs in themselves and their communities in a hyper-aged society. Their activities are significant in that they are working to extend the healthy life expectancy of their communities. They may therefore provide a model for other countries with aging populations, and the development of a scale to measure their competency is likely to be important to the international community.

We developed a scale consisting of behavioral and perceptual aspects to assist in grading the competencies of CHWs working to address NCDs. In turn, this tool may assist community health program policy makers and managers as they design and improve these programs. The concept of competency was proposed by Spencer and Spencer in 1993. Studies of competency tend to focus on highly productive workers, examining the mechanisms that enable them to achieve results. The competencies of high performers have unique characteristics, including behavioral aspects (e.g., skills and knowledge) and perceptual aspects (e.g., attitudes and values) [29–31]. The concept of competency therefore needs to be structured in terms of both perception and behavior. Measuring behavioral aspects can help to identify how CHWs’ efforts can be integrated into the health system, and how their inputs can be combined with social support to achieve a greater return on investment. Measuring perceptual aspects could be useful for considering fair payment for labor and how to prevent disturbance or interference with CHWs’ position in the community. By measuring the competencies of CHWs, we can consider what perceptual and behavioral programs (including new training and mentoring programs) to introduce when training or re-training CHWs. As a result, CHWs may be more likely to see preventing NCDs as a challenging community health problem to be resolved, rather than a threat to be avoided, and can also set challenging goals and demonstrate a stronger role in practice. In turn, this could contribute to extending healthy life expectancy and reducing health disparities. However, at present, there are no scales for measuring the perceptual and behavioral competency of CHWs in NCD prevention and control.

This study aimed to develop a CHWs’ perceptual and behavioral competency scale for preventing NCDs (COCS-N) in a developed country, and to assess the reliability and validity of the scale.

## Methods

### Phase 1: developing the instrument

A first draft of the COCS-N was developed following a critical review of the relevant literature. Articles were identified in PubMed and Ichushi-Web on the theme of competency among CHWs. The following search terms were used: “community health workers”, “community health volunteers”, “health promotion volunteers”, and “competency”, along with the MeSH terms “adult”, “middle-aged”, and “aged”. These searches yielded eight articles [32–39]. The inclusion of an article for analysis was determined by two criteria: 1) it was related to research on the perceptions and behaviors of CHWs; and 2) it was associated with existing competency scales. Four additional articles were included that dealt with measures related to the four roles of CHWs [40–43]. On the basis of a literature review and the researchers’ experience, a first draft of the COCS-N was constructed containing 30 items.

To ensure the content validity of the first draft of the COCS-N, 15 experts were invited to rate the relevance of each item in relation to the perceptual and behavioral competency of CHWs. The experts included two experienced researchers (professors), four public health nurses who support CHWs, and nine CHWs in two local government areas. We then revised the first draft of the COCS-N based on these experts’ opinions (e.g., to avoid complex questions and ambiguous wording). The revised COCS-N included 20 items.

### Phase 2: validating the instrument

#### Participants and settings

We conducted a survey among 1743 local governments in Japan, and eligible participants were selected between July 2020 and September 2020. We confirmed the existence of CHWs and consent for research cooperation with all local governments. Before distributing the survey questionnaires, we sent informed consent letters to the administrators of all local governments in Japan; of these, 194 (11.1%) consented to participate in the study. The survey questionnaires were distributed to each participant by staff from the local government. The inclusion criteria for individual participants were as follows: 1) participation in health promotion activities for at least 1 year; and 2) participation in the survey, as determined by public health nurses supporting them. We excluded anyone who had participated in health promotion activities for less than 1 year because competency in community health work is developed through practical application. Previous studies have indicated that there are perceptions and behaviors that can only be acquired through actual relationships with local residents. If a potential participant had engaged in health promotion activities for less than 1 year, we considered it unlikely that they had acquired sufficient competency as a CHW, and we were concerned that this would affect the accuracy of the data. Data were collected between September and November 2020.

#### Measures

Participants’ demographic information included age, sex, residential area, number and type of diseases currently being addressed, co-habiting family situation, and number of years as a CHW.

All items in the COCS-N were scored on a four-point Likert-type scale ranging from 0 (disagree) to 3 (agree). To assess the concurrent validity of the scale, participants were also asked to complete each item in the Japanese versions of the European Health Literacy Survey Questionnaire (HLS-EU-Q47) [44, 45] and the community commitment scale (CCS) [46]. The HLS-EU-Q47 consists of 47 items that measure health literacy using a four-point Likert-type scale. Responses were scored as follows: 1 = very difficult, 2 = fairly difficult, 3 = fairly easy, 4 = very easy. Total scores ranged from 47 to 188. High scores on this scale indicate a high level of health literacy. For this scale, Cronbach’s alpha coefficient was 0.97. The CCS consists of eight items, rated on a four-point Likert-type scale, which measure socializing in and belonging to a community (i.e., community commitment). Responses are scored from 0 = strongly disagree to 4 = strongly agree; the total score ranges from 0 to 32. High scores on this scale indicate a high level of community commitment. For this scale, Cronbach’s alpha coefficient was 0.78. Previous studies reported that CHWs’ competency includes aspects of their roles as both transmitters of health information and local residents. We predicted that high levels of health literacy and community commitment, as indicated by higher HLS-EU-Q47 and SSC scores, would reflect a desire to acquire competency, as indicated by higher COCS-N scores. We therefore used these scales as an indicator of concurrent validity.

### Statistical analyses

We used IBM SPSS ver. 24.0 and Amos 27.0 (IBM Corp., Armonk, NY, USA) for all statistical analyses. An item analysis and exploratory factor analysis were used to evaluate the reliability and convergent validity of the initial COCS-N version, referring to previous studies [47]. The statistical analysis was supervised by an expert in quantitative research. The criteria for the item analysis included rate-of-response difficulty (non-respondents: ≥ 5%), distribution (one specific answer in a four-point Likert-type scale: ≥ 95%), skewness and kurtosis (absolute values of < 1.0 each), correlations of each item (correlation coefficient: > 0.6), a good–poor analysis (no significant differences between the highest-scoring and lowest-scoring groups), and an item–total analysis (correlation coefficient between the item and the total score without that item: ≥ 0.5). After the item analysis, the total sample was randomly divided into two for cross-validation. An exploratory factor analysis (EFA) was used for Group 1, and a confirmatory factor analysis (CFA) was used for Group 2.

To assess the dimensionality of the COCS-N, an EFA (maximum likelihood solution method) with promax rotation was performed on the development sample. Dimensionality was assessed using an eigenvalue > 1.0 and a scree plot. Item loading had to exceed 0.40. A CFA was then used to verify the construct validity of the scale. The goodness-of-fit index (GFI), adjusted goodness-of-fit index (AGFI), comparative fit index (CFI), and root mean square error of approximation (RMSEA) were used to evaluate the data–model fit. The model was accepted if the GFI, AGFI and CFI were ≥ 0.900 and the RMSEA was ≤0.050. Criterion-related validity was examined using the total scores for the HLS-EU-Q47 and the CCS.

Internal consistency and reliability were evaluated by calculating Cronbach’s alpha coefficient for the COCS-N, with alpha ≥0.70 considered acceptable.

## Results

### Demographic characteristics

In total, 6480 individuals returned the questionnaire, 3651 (56.3%) of whom met the inclusion criteria. A further 531 individuals were excluded because of insufficient participation in health promotion activities (i.e., less than 1 year; *n* = 17), no response to the COCS-N or CCS (*n* = 268), or missing responses for two or more items of the HLS-EU-Q47 (*n* = 246). This left 3120 participants for inclusion in the analysis. Table 1 shows the participating CHWs’ demographic information. Participants ranged in age from 24 to 93 years, with an average age of 67.0 years (standard deviation [SD] = 9.0 years), and 88.8% were female. The number of people living in the households of CHWs ranged from 0 to 6, with an average of 1.5 (SD = 1.0). The number of different diseases that CHWs were currently addressing ranged from 0 to 9, with an average of 0.9 (SD = 1.0). The length of time that participants had been working as CHWs ranged from 1 to 57 years, with an average of 8.2 years (SD = 7.7 years).

**Table 1:** Demographic characteristics of community health workers

*n* = 3120		
	Number or Mean ± SD^a^	% or (Range)
Age	67.0 ± 9.0	(24–93)
< 25	2	0.1
25–34	10	0.3
35–44	58	1.9
45–54	225	7.2
55–64	685	22.0
65–74	1435	46.0
≥ 75	533	17.1
Missing	24	0.8
Sex		
Female	2771	88.8
Male	338	10.8
Missing	11	0.4
Area of residence		
Chubu	802	25.7
Kinki	686	22.0
Kanto	485	15.5
Tohoku	465	14.9
Kyusyu/Okinawa	396	12.7
Chugoku/Shikoku	240	7.7
Hokkaido	38	1.2
Missing	11	0.4
People living together	1.5 ± 1.0	(0–6)
Living with spouse	1184	37.9
Living with spouse & children	622	19.9
Living alone	361	11.6
Living together with three generations	419	13.4
Living with children	184	5.9
Others	349	11.2
Missing	1	0.0
Diseases under treatment	0.9 ± 1.0	(0–9)
Type of disease		
Hypertension	918	29.4
Visual impairment	338	10.8
Musculoskeletal diseases	313	10.0
Diabetes mellitus	271	8.7
Heart disease	126	4.0
Other	510	16.3
Missing	28	0.9
Years as a CHW	8.2 ± 7.7	(1–57)
Missing	57	1.8

^a^*SD* standard deviation

### Item analysis

Table 2 shows the item analysis results. Items 1, 2, 11, and 12 were excluded based on the population distribution. Item 9 was excluded based on the skewness and the item–total analysis. The correlation coefficient between items 18 and 19 was higher than 0.6. Item 18 was excluded based on the item–total analysis. Exploratory factor analysis with promax rotation was then performed for the remaining 14 items.

**Table 2:** Item analysis of “the community health workers perceptual and behavioral competency scale for preventing non-communicable diseases”

*n* = 3120											
No.	Item	Item difficulty^a^	Population distribution^b^				Kurtosis / Skewness^c^		Correlation of item^d^	Good-Poor analysis^e^	Item-Total correlation^f^
			0	1	2	3					
1	The activities of a community health worker bring interest to my life and make it feel worthwhile.	1.0	1.4	16.8	55.6	26.1	−0.093	− 0.343	–	.000^**^	.657^**^
2	What I do as a community health worker makes me aware of the importance of my own health.	0.6	0.3	3.3	40.4	56.0	0.105	−0.808	–	.000^**^	.562^**^
3	I feel that my family and neighbors support my activities as a community health worker.	0.8	4.9	31.7	46.0	17.4	−0.499	−0.135	–	.000^**^	.589^**^
4	I find that I enjoy what I do as a community health worker because I can learn new things about health.	1.0	0.4	7.9	47.6	44.1	−0.18	−0.575	–	.000^**^	.622^**^
5	I feel motivated to convey information and advice on healthy lifestyles to my family and neighbors.	0.6	0.6	9.3	54.2	35.8	−0.026	−0.432	–	.000^**^	.692^**^
6	I’m happy to see that other people are pleased with my activities as a community health worker.	0.7	0.9	12.5	44.7	41.8	−0.272	− 0.606	–	.000^**^	.669^**^
7	I enjoy the time I spend with the local people, helping them enhance their health.	0.7	1.3	16.7	48.4	33.6	−0.407	− 0.428	–	.000^**^	.717^**^
8	I want to work with the local people to maintain and improve everyone’s health as much as possible.	0.5	0.6	8.1	46.9	44.4	−0.05	− 0.628	–	.000^**^	.693^**^
9	I trust the professionals (public health nurses, nutritionists, etc.) who are supporting the activities of community health workers.	0.5	0.1	4.0	34.5	61.4	0.231	−1.006	–	.000^**^	.472^**^
10	I like and respect the colleagues I work with, such as the community health workers whose team I’m on.	0.7	0.4	6.3	40.3	53.0	0.179	−0.850	–	.000^**^	.560^**^
11	I can try out beneficial lifestyle habits myself that I have found out about through my activities as a community health worker.	1.0	0.3	9.4	56.4	33.9	−0.269	−0.29	–	.000^**^	.643^**^
12	I can collect well-grounded scientific information on health.	0.9	1.0	16.9	56.2	25.9	−0.222	−0.27	–	.000^**^	.575^**^
13	I can incorporate seasonal food ingredients into daily meals and share the enjoyment of eating them with my family and neighbors.	0.9	1.6	17.7	52.4	28.2	−0.25	−0.366	–	.000^**^	.554^**^
14	I can recommend physical exercises and sports to my family and neighbors, and invite them to take part in them.	0.6	3.9	26.3	44.5	25.3	−0.601	−0.270	–	.000^**^	.699^**^
15	I can convey to my family and neighbors the importance of eating well-balanced meals.	0.8	3.1	24.2	52.0	20.7	−0.269	−0.278	–	.000^**^	.658^**^
16	I can teach physical exercises and sports to my family and neighbors that they can easily incorporate into their daily lives.	0.9	6.3	32.6	43.7	17.4	−0.562	−0.128	–	.000^**^	.681^**^
17	I can talk about health to local people at sites of community gatherings.	0.9	7.4	32.2	43.2	17.3	−0.578	−0.152	–	.000^**^	.687^**^
18	I can communicate to professionals (doctors, public health nurses, etc.) the health concerns that the local people have.	0.9	8.2	37.1	39.2	15.5	−0.635	−0.014	+	.000^**^	.610^**^
19	I can share, with professionals (public health nurses, nutritionists, etc.), information about the health challenges that the community faces.	1.0	6.2	32.2	45.6	16.0	−0.475	−0.152	+	.000^**^	.625^**^
20	I can cooperate with area organizations and engage in programs to promote people’s health.	0.7	2.9	19.0	53.0	25.1	−0.077	−0.421	–	.000^**^	.679^**^

Exclusion criteria for the item analysis

^a^The proportion of items with no answers was over 5% of the sample

^b^Items with a score (0 to 3) of 55% or higher in the sample

^c^Absolute value of skewness or kurtosis was less than −1 or greater than 1

^d^Correlation was over 0.6

^e^Difference in the average score between the highest scoring group and the lowest scoring group was not significant (*P* ≥ 0.05)

^f^The correlation coefficient between the item and the total of all the items (not including the item itself) was 0.5 or lower

^**^P < 0.001

### Factor structure

The results of exploratory factor analysis are shown in Table 3. The scree plots showed a sharp slope between factors one and two and between factors two and three. The eigenvalues were 6.091 for factor one, 1.261 for factor two, and 0.691 for factor three. Eigenvalues and scree plots suggested a one- or two-factor model. The promax rotation was repeated based on a factor loading of 0.4. At the initial stage, items 3 and 20, which did not have a factor loading of 0.4, were deleted. The analysis was repeated and the optimal solution was obtained when items 5, 10, 13 and 14 were deleted, in addition to items 3 and 20. The final scale was extracted as eight items with two factors. Factor 1 included four items (items 7, 4, 8, and 6) interpreted as “Sharing the pleasure of living a healthy life”, reflecting CHWs’ perceptions on health promotion activities. Factor 2 included four items (items 17, 16, 19, 15), interpreted as “Creating healthy resources”, reflecting CHWs’ behavior in carrying out health promotion activities. The factor loadings were greater than 0.4 for each factor. Cumulatively, the two factors explained 68.9% of the variance. The correlation coefficient between the two factors was 0.61.

**Table 3:** Exploratory factor analysis of “the community health workers perceptual and behavioral competency scale for preventing non-communicable diseases”

*n* = 1560				
No.	Item	Factor I	Factor II	Total scale communality
		“Sharing the pleasure of healthy life”	“Creating the healthy resources”	
7	I enjoy the time I spend with the local people, helping them enhance their health.	**0.861**	0.032	0.78
4	I find that I enjoy what I do as a community health worker because I can learn new things about health.	**0.861**	−0.118	0.63
8	I want to work with the local people to maintain and improve everyone’s health as much as possible.	**0.799**	0.080	0.72
6	I’m happy to see that other people are pleased with my activities as a community health worker.	**0.771**	0.074	0.67
17	I can talk about health to local people at sites of community gatherings.	−0.025	**0.885**	0.76
16	I can teach physical exercises and sports to my family and neighbors that they can easily incorporate into their daily lives.	0.040	**0.819**	0.71
19	I can share, with professionals (public health nurses, nutritionists, etc.), information about the health challenges that the community faces	−0.065	**0.815**	0.60
15	I can convey to my family and neighbors the importance of eating well-balanced meals.	0.098	**0.737**	0.64
Cumulative contribution (%)		56.1	68.9	
Factor correlation coefficients (r)	Factor I	1.00	0.61	
	Factor II	0.61		

Maximum likelihood solution method with promax rotation; missing data were excluded

Bold: Item loadings exceed 0.40

### Internal consistency and validity

**Fig. 1:**
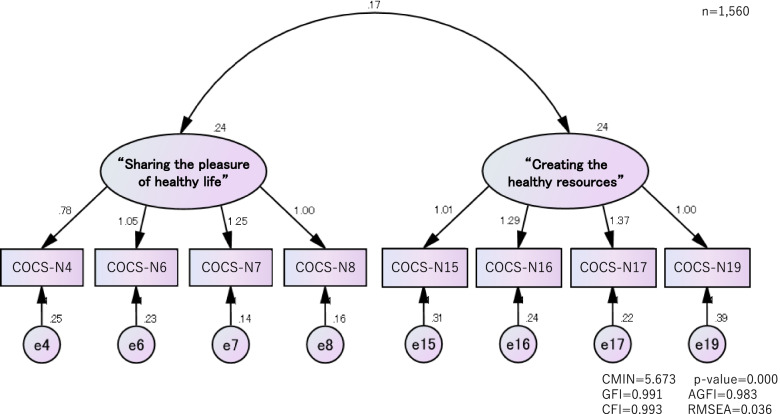
Confirmatory factor analysis of “the community health workers perceptual and behavioral competency scale for preventing non-communicable diseases” (final version)

Cronbach’s alpha coefficients were 0.83 for Factor 1, 0.81 for Factor 2, and 0.87 for the overall scale. The two factors were entered as latent factors in a confirmatory factor analysis model. In the initial model, GFI = 0.991, AGFI = 0.983, CFI = 0.993, and RMSEA = 0.036, suggesting a good data–model fit (Fig. 1).

Pearson’s correlation analysis revealed correlations between the total COCS-N score and the total score of both the HLS-EU-Q47 and the CCS. COCS-N scores were strongly positively correlated with HLS-EU-Q47 scores (*r* = 0.577; *P* < 0.001), and showed a moderate correlation with CCS scores (*r* = 0.447; *P* < 0.001).

## Discussion

In public health, CHWs play an important role in disease prevention and health promotion, by bridging the gap between the community and public health professionals. However, scales for measuring the competency of CHWs are currently lacking. To the best of our knowledge, the COCS-N is the first scale developed for this purpose. The COCS-N demonstrated adequate reliability (Cronbach’s alpha: 0.87) and validity (*r* = 0.577; *P* < 0.001 with the HLS-EU-Q47 scale; *r* = 0.447; *P* < 0.001 with the CCS scale). The dimensionality was confirmed by the CFA, which indicated a good fit (GFI = 0.991, AGFI = 0.983, CFI = 0.993, RMSEA = 0.036). The proportion of respondents was low (56.3%). The demographic characteristics of non-responders were unknown, so the sample may have been biased. However, our response rate was similar to that of a previously published study polling the same population [48]. Most of the participating CHWs were women (88.8%), with an average age of 67.0 years (SD = 9.0). According to both an official evaluation by the Japanese government and a previous study, this demographic profile is similar to that of participants in a previous survey of CHWs [11, 49]. The sample was therefore considered to be representative of the population of CHWs in Japan. Participants in this study had all been actively working as CHWs for at least 1 year, to increase the accuracy of measurement of competencies. However, we believe that this scale could be used for any CHW, from novice to expert. The scale can also be used regularly (e.g., every 6 months) to track the development of CHWs’ competences. We suggest that its development and testing in Japan mean that the tool may also be applicable to other high-income settings.

The proposed scale can measure both perceptual and behavioral aspects of CHWs’ competency, and can clarify the relationship between perception and behavior among CHWs’ health promotion activities. The first factor of the COCS-N, “Sharing the pleasure of living a healthy life”, covered the perceptional dimension and included four items: “I find that I enjoy what I do as a health promoter because I can learn new things about health”; “I’m happy to see that other people are pleased with my activities as a community health worker”; “I enjoy the time I spend with local people, helping them enhance their health”; and “I want to work with local people to maintain and improve everyone’s health as much as possible”. These items sought to capture the nuances of CHWs’ perception that healthy living and positive health behaviors can be pleasurable activities, and that they share that perception with others. The health behavior model sees health beliefs and perceptions, such as awareness of the likelihood of contracting a particular disease, as a prerequisite for positive health behaviors [50]. The factor “Sharing the pleasure of living a healthy life” can therefore be used to evaluate the perceptions of CHWs that contribute to their ability to promote healthy values to their communities and motivate others to engage in healthy behaviors. The results indicate that the sense of enjoyment of healthy living, and the value to health in sharing this enjoyment, may be a non-monetary and non-material reward for CHWs. We also consider that the amount and content of work that can be done with a sense of enjoyment should be taken into account, so as not to hinder or obstruct the position of CHWs in the community.

The second factor of the COCS-N, “Creating healthy resources”, covered behavioral issues. It consisted of four items: “I can convey to my family and neighbors the importance of eating well-balanced meals”; “I can teach physical exercises and sports to my family and neighbors that they can easily incorporate into their daily lives”; “I can talk about health to local people at community gatherings”; and “I can share information about the health challenges that the community faces with professionals (e.g., public health nurses and nutritionists)”. These items captured the behaviors that can help strengthen resources for community members, and enable them to incorporate healthy behaviors into their daily lives. Theories about health behavior and behavioral change make clear that behavioral change requires both intrinsic factors and resources that help people implement a given behavior [51, 52]. Health resources may be at both the individual and community levels. Creating individual health resources is related to integrating efforts into the health system, and creating community health resources is related to increasing the effectiveness of other material support and combinations. There is some evidence that individual healthy behavior is determined by social group and community [53, 54]. Assessing behavior among CHWs that helps create healthy resources can therefore be useful for evaluating behaviors that help to align external factors, such as the acquisition of resources that facilitate the implementation of health behaviors.

This study had several limitations. First, the statistical analyses were not tested for sources of bias or confounding that could affect the results. For example, the competencies of CHWs could be influenced by population size, age, sex, severity and comorbidity of NCDs in their community, and other community social resource factors. Bias may also have been created because access to the target population was difficult during the COVID-19 pandemic, and therefore only 11.1% of municipalities agreed to participate. Future research needs to take into account these potential biases and their influence. Second, the cross-sectional design means that it is not possible to make valid judgments about the direct relationship between COCS-N scores and factors such as the incidence and morbidity of NCDs in communities. A prospective design is needed to determine the predictive validity of this measure.

## Conclusion

The COCS-N was developed to measure the competencies of CHWs in delivering preventive care for NCDs to local populations, where it can be difficult for professionals to intervene. This scale is a reliable and valid instrument. We demonstrated that the concept of competency for CHWs consists of perceptions about sharing the pleasure of living a healthy life and behavioral aspects around creating healthy resources. The COCS-N measures the perceptual and behavioral competency of CHWs in their daily activities, providing a scale for measuring the competencies of CHWs working on NCD prevention. This scale can be used to clarify the competencies that CHWs have acquired, and to identify practical skills that could be further developed through training. Enhancement of the two aspects of CHWs’ competency should increase the effectiveness of NCD prevention in communities. This, in turn, can contribute to extending healthy life expectancy and reducing health disparities.

## Supplementary Information

**Additional file 1.** The COCS-N English Version

Additional file 2. The COCS-N Japanese Version

## Data Availability

The datasets generated and analyzed during this study are not publicly available because the Ethical Guidelines for Epidemiological Research by the Japanese Government and the National Basic Resident Registration System administered by the Ministry of Internal Affairs and Communications in Japan prohibit researchers from providing their research data to other third-party individuals. However, they are available from the corresponding author on reasonable request.

## Abbreviations

AGFI: Adjusted goodness-of-fit index
CCS: Community commitment scale
CFA: Confirmatory factor analysis
CFI: Comparative fit index
CHWs: Community health workers
COCS-N: Community Health Workers Perceptual and Behavioral Competency Scale for Preventing Non-Communicable Diseases
EFA: Exploratory factor analysis
GFI: Goodness-of-fit index
HLS-EU-Q47: European Health Literacy Survey Questionnaire
NCD: Non-communicable disease
RMSEA: Root mean square error of approximation
