# Supplementary material for: Development of a community health workers perceptual and behavioral competency scale for preventing non-communicable diseases (COCS-N) in Japan
Source: BMC Public Health. 2022 Jul 26;22:1416. doi: 10.1186/s12889-022-13779-5 (PMC9315843; doi:10.1186/s12889-022-13779-5)
Supplement: Supplementary file 1 — Additional file 1. The COCS-N English Version [file 12889_2022_13779_MOESM1_ESM.pdf]

Additional file 1. The COCS-N English Version

A Community Health Workers Perceptual and Behavioral Competency Scale for Preventing Non-Communicable Diseases (COCS-N), English Version

Please circle (○) the number that you could say, "most closely matches your thought and situation for each statement".

No	Item	Disagree	Disagree somewhat	Agree somewhat	Agree
1	I enjoy the time I spend with the local people, helping them enhance their health.	0	1	2	3
2	I find that I enjoy what I do as a health promoter because I can learn new things about health.	0	1	2	3
3	I want to work with the local people to maintain and improve everyone's health as much as possible.	0	1	2	3
4	I'm happy to see that other people are pleased with my activities as a health promoter.	0	1	2	3
5	I can talk about health to local people at sites of community gatherings.	0	1	2	3
6	I can teach physical exercises and sports to my family and neighbors that they can easily incorporate into their daily lives.	0	1	2	3
7	I can share, with professionals (public health nurses, nutritionists, etc.), information about the health challenges that the community faces.	0	1	2	3
8	I can convey to my family and neighbors the importance of eating well-balanced meals.	0	1	2	3

Imamatsu Y, Tadaka E, Development of a Community Health Workers Perceptual and Behavioral Competency Scale for Preventing Non-Communicable Diseases (COCS-N) in Japan
