# Supplementary material for: Development of a community health workers perceptual and behavioral competency scale for preventing non-communicable diseases (COCS-N) in Japan
Source: BMC Public Health. 2022 Jul 26;22:1416. doi: 10.1186/s12889-022-13779-5 (PMC9315843; doi:10.1186/s12889-022-13779-5)
Supplement: Supplementary file 2 — The COCS-N Japanese Version [file 12889_2022_13779_MOESM2_ESM.pdf]

Additional file 2. The COCS-N Japanese Version

A Community Health Workers Perceptual and Behavioral Competency Scale for Preventing Non-Communicable Diseases (COCS-N), Japanese Version

コミュニティ・ヘルス・ワーカーズ（健康推進員等）の認知・行動能力尺度（COCS–N），日本語版

各項目について、あなたの現在のお考えや状況に最も近い数字1つに○をつけて下さい。

No	Item	あてはまる	ややあてはまる	ややあてはまらない	あてはまらない
1	私は、地域の人たちと健康づくりに取り組む時間が楽しい	0	1	2	3
2	私は、推進員の活動で健康に関する新しいことが学べて楽しい	0	1	2	3
3	私は、地域の人たちと一緒に少しでも健康を維持したり伸ばしたりしていきたい	0	1	2	3
4	私は、推進員の活動によって相手が喜んでくれることが嬉しい	0	1	2	3
5	私は、地域の集まりの場で、地域の人たちに対して健康について話すことができる	0	1	2	3
6	私は、家族や近隣住民に、生活の中に取り入れやすい体操や運動を教えることができる	0	1	2	3
7	私は、「地域の健康課題」について、専門職（保健師・栄養士等）と情報を共有できる	0	1	2	3
8	私は、家族や近隣住民にバランスの良い食事の重要性を伝えることができる	0	1	2	3

Imamatsu Y, Tadaka E, Development of a Community Health Workers Perceptual and Behavioral Competency Scale for Preventing Non-Communicable Diseases (COCS-N) in Japan
